# The Hedonic and Eudaimonic Motives for Activities: Measurement Invariance and Psychometric Properties in an Adult Japanese Sample

**DOI:** 10.3389/fpsyg.2020.01220

**Published:** 2020-06-10

**Authors:** Ryosuke Asano, Tasuku Igarashi, Saori Tsukamoto

**Affiliations:** ^1^Department of Psychology, Kurume University, Kurume, Japan; ^2^Graduate School of Education and Human Development, Nagoya University, Nagoya, Japan; ^3^Division of Liberal Arts and Sciences, Aichi Gakuin University, Nisshin, Japan

**Keywords:** well-being, Hedonic and Eudamonic Motives for Activities, measurement invariance, reliability, validity, adults, Japan

## Abstract

Hedonic pleasure orientation (seeking enjoyment), hedonic relaxation orientation (seeking comfort), and eudaimonic orientation (seeking meaning) are major ways that people pursue well-being. We investigated the measurement invariance and psychometric properties of the Hedonic and Eudamonic Motives for Activities (HEMA) scale in a Japanese adult sample (*N* = 1,892). The Japanese HEMA scale demonstrated measurement invariance at the configural, metric, scalar, and strict levels across gender and age groups. Latent mean differences of the scale across these demographic groups were less than small. The scale showed high internal consistency and six-week test-retest reliability and reasonable correlations with life satisfaction, positive affect, negative affect, psychological well-being, and interdependent happiness. In sum, these findings suggest that the Japanese HEMA scale is useful to capture hedonic and eudaimonic conceptions of well-being as orientations. It is hoped that our findings will stimulate further research on well-being using the HEMA scale.

## Introduction

Research on well-being has two different, yet overlapping, perspectives: hedonism and eudaimonism. The hedonic perspective concerns the pursuit of pleasant and comfortable states, while the eudaimonic perspective concerns living a good life and being fully functioning (see, for a review, Ryan and Deci, 2001; Ryan et al., 2008). Huta and Waterman (2014; see also Huta, 2016) summarized the literature regarding well-being from these two perspectives in terms of four categories: orientations, behaviors, experiences, and functioning. Of these, orientations represent motives, values, and goals. Orientations shape the direction of a person’s actions and are thus more fundamental than behaviors representing their specific actions. Compared to experiences (e.g., life satisfaction, positive affect, and lack of negative affect) and functioning (e.g., psychological well-being), orientations stem more from personal choice, which can be changed if desired or necessary. The above discussions suggest that orientations can provide a better definition of well-being than the other categories. In this study, we focus on orientations to define well-being from the hedonic and eudaimonic perspectives.

The Hedonic and Eudamonic Motives for Activities (HEMA; Huta and Ryan, 2010) scale is used to measure both the hedonic and eudaimonic conceptions of well-being as orientations. The HEMA scale has been translated into many languages, including German, Swedish, Polish, Italian, and Japanese (see, for a review, Huta, 2016). Although the HEMA scale was originally developed to operationalize hedonic and eudaimonic orientations (Huta and Ryan, 2010), recent evidence has shown that the hedonic orientation of the scale can be divided into two different components (Asano et al., 2014, 2018; Bujacz et al., 2014; Braaten et al., 2019). “Hedonic pleasure” and “hedonic relaxation” orientations refer to striving to feel enjoyment and comfort, respectively. “Eudaimonic” orientation refers to striving to do what is meaningful, even if difficult to achieve. Research on the Japanese HEMA scale with student samples demonstrated that the three-factor model was better than the two-factor model and showed that the three subscales were adequately reliable and valid (Asano et al., 2014, 2018).

This study extended earlier findings on the Japanese HEMA scale in two ways. First, we investigated whether the factor structure of the Japanese HEMA scale is equivalent across different demographic groups (i.e., gender and age) with an adult sample. No studies have yet tested measurement invariance across gender and age groups, although past research reported that the HEMA scale had little relationship with gender and age (Huta, 2016). Because gender and age are potential individual characteristics that may influence scale ratings of well-being (e.g., Emerson et al., 2017), it is crucial to test the equivalence of the factor structure of the scale for these demographic factors. Therefore, we assessed measurement invariance and latent means of the Japanese HEMA scale across gender and age groups.

Second, we added evidence for the reliability and validity of the Japanese HEMA scale in an adult sample. The scale has revealed acceptable internal consistency and temporal stability over four- and eight-week periods in student samples (Asano et al., 2014, 2018). Thus, we expected that the three Japanese HEMA subscales would indicate high internal consistency measured by Cronbach’s alpha and McDonald’s omega (>0.80) and moderate six-week temporal stability measured by test-retest intraclass correlation coefficient (ICC; >0.50). In addition, this study examined associations of the Japanese HEMA scale with outcome variables, such as life satisfaction, positive and negative affect, and psychological well-being. Based on the previous findings (Asano et al., 2014, 2018), we expected that hedonic pleasure and eudaimonic orientations would be associated more strongly with life satisfaction and positive affect than hedonic relaxation orientation. We also expected that hedonic pleasure and eudaimonic orientations would be weakly associated with negative affect. Furthermore, we expected that eudaimonic orientation would be associated most strongly with psychological well-being, followed by the hedonic pleasure and hedonic relaxation orientations, because eudaimonic orientation related more strongly to personal growth, sense of meaning, and self-actualization than the two hedonic orientations (Huta and Ryan, 2010; Asano et al., 2014, 2018; Braaten et al., 2019). Besides these Western-driven outcome variables, it is instructive to assess variables that are valued in Eastern context, particularly in Japan. Therefore, our study included interdependent happiness that can be achieved through interpersonal harmony (Hitokoto and Uchida, 2015) as an outcome variable.

The present research explored the measurement invariance and psychometric properties of the Japanese HEMA scale in a large adult sample. First, we tested gender and age invariance for the three-factor structure of the scale. We also investigated the latent mean differences across gender and age groups. Second, we expected that the scale would indicate adequate internal consistency, six-week temporal stability, and criterion validity. Five scales were used as outcome variables: life satisfaction, positive affect, negative affect, psychological well-being, and interdependent happiness.

## Materials and Methods

### Participants

The present study was approved by the Institutional Review Board at Kurume University (Protocol No. 310). Data came from 2,100 Japanese residents aged 20 or older, recruited by a marketing research firm, Cross Marketing Inc. Six weeks later, they were contacted for a follow-up assessment. We excluded 116 (5.5%) participants at the initial assessment and 92 (4.6%) participants at the follow-up assessment due to failure on attention check items (“Please choose answer ‘strongly agree’ to this item;” Maniaci and Rogge, 2014).

The final sample consisted of 1,892 Japanese adults (865 males, 1,027 females; *M*_age_ = 50.28 ± 14.42 years). The sample was split into three age groups: 595 were aged between 20 and 39 years (252 males, 343 females; *M*_age_ = 33.06 ± 4.83 years); 628 were aged between 40 and 59 years (288 males, 340 females; *M*_age_ = 49.71 ± 5.66 years); and 669 were aged between 60 and 79 years (325 males, 344 females; *M*_age_ = 66.14 ± 4.61 years). There were no missing values for all variables in the first wave. Of the final sample, 80.2% (*n* = 1,517) participated in the follow-up assessment for the Japanese HEMA scale. Compared to those who failed to participate in the follow-up assessment, participants with complete data were slightly higher in hedonic pleasure orientation (*d* = 0.19), slightly more likely to be female (*V* = 0.07), and younger (*d* = 0.26).

### Measures

#### Hedonic and Eudaimonic Motives for Activities

The Japanese HEMA scale includes 11 items covering the range of well-being as orientations (Asano et al., 2014, 2018). Asano et al. (2014) translated the original nine items (Huta and Ryan, 2010) into Japanese and back-translated them to check for language equivalence. The current Japanese HEMA scale includes two additional items regarding the hedonic relaxation orientation (see Table 2 for the exact items). The instructions were “To what degree do you typically approach your activities with each of the following intentions, whether or not you actually achieve your aim?” Participants rated each item on a 7-point scale (1 = *not at all*, 7 = *very much*).

#### Outcome Variables

Life satisfaction was measured using the five-item Satisfaction with Life Scale (Diener et al., 1985), translated into Japanese by Oishi (2009). Sample items include “In most ways, my life is close to my ideal” and “The conditions of my life are excellent.” Participants rated each item on a 7-point scale (1 = *strongly disagree*, 7 = *strongly agree*; *M* = 18.07, *SD* = 6.27, and α = 0.91).

Positive and negative affect were measured using Mroczek and Kolarz’s (1998) six-item (each) scale, translated into Japanese in the Midlife Development in Japan (MIDJA; Ryff et al., 2018). Sample positive affect items include “cheerful” and “calm and peaceful.” Sample negative affect items include “nervous” and “worthless.” Participants rated how much of the time during the past 30 days they felt each emotion on a 5-point scale (1 = *none of the time*, 5 = *all of the time*; *M* = 18.39, *SD* = 4.40, α = 0.92 for positive affect; *M* = 13.59, *SD* = 4.43, and α = 0.85 for negative affect).

Psychological well-being was measured using Ryff and Keyes’s (1995) 18-item scale, translated into Japanese in the MIDJA study (Ryff et al., 2018). Sample items include “I think it is important to have new experiences that challenge how you think about yourself and the world” and “Some people wander aimlessly through life, but I am not one of them.” Participants rated each item on a 7-point scale (1 = *strongly disagree*, 7 = *strongly agree*; *M* = 78.48, *SD* = 10.34, and α = 0.78).

Interdependent happiness was measured using the nine-item Interdependent Happiness Scale (Hitokoto and Uchida, 2015). Sample items include “I make significant others happy” and “I believe that my life is just as happy as that of others around me.” Participants rated each item on a 5-point scale (1 = *strongly disagree*, 5 = *strongly agree*; *M* = 28.65, *SD* = 6.66, and α = 0.91).

### Data Analysis

We employed multi-group confirmatory factor analysis with maximum likelihood robust estimation to test the gender and age invariance of the Japanese HEMA scale. We compared four nested models: configural, metric, scalar, and strict. Configural invariance confirms that the same factor structure occurs across groups as the baseline model. Metric invariance means the same factor loadings occur across groups. Scalar invariance means the same item intercepts occur across groups. Strict invariance means the same item residual variances occur across groups. Given that χ^2^ is sensitive to sample size, values of comparative fit index (CFI) ≥ 0.90, root mean square error of approximation (RMSEA) ≤ 0.08, and standardized root mean square residual (SRMR) ≤ 0.08 were considered indicators of acceptable fit (Brown, 2015). The fit of nested models was evaluated using a worsening of CFI (ΔCFI) less than or equal to 0.01 and a worsening of RMSEA (ΔRMSEA) less than or equal to 0.015 (Chen, 2007). Mplus 8.3 (Muthén and Muthén, 1998-2019) was used for the analysis.

If scalar or strict invariance was observed, we proceeded with comparing latent factor means of the Japanese HEMA scale across gender and age groups. The latent means in male, 20–39 years, and 40–59 years, respectively, were set to zero (i.e., these subgroups functioned as the reference groups), whereas the latent means in the remaining groups were freely estimated. We interpreted Cohen’s *d* effect sizes of 0.20, 0.50, and 0.80 as small, moderate, and large, respectively (Cohen, 1992).

We then examined internal consistency (Cronbach’s alpha and McDonald’s omega), temporal stability (test-retest ICC), and criterion validity (correlations with outcome variables) of the Japanese HEMA scale. We considered correlation coefficients of 0.10, 0.20, and 0.30 as small, moderate, and large, respectively (Gignac and Szodorai, 2016). R 3.5.0 (R Core Team, 2018) was used for the analysis.

## Results

Confirmatory factor analysis conducted on the whole sample indicated that the three-factor model [χ^2^ (41) = 473.23, *p* < 0.001, CFI = 0.937, RMSEA = 0.075 (90% CI = 0.069, 0.081), and SRMR = 0.051] was better than the two-factor model of the Japanese HEMA scale [χ^2^ (43) = 1182.52, *p* < 0.001, CFI = 0.834, RMSEA = 0.118 (90% CI = 0.113, 0.124), and SRMR = 0.087]. The inter-factor correlations were 0.76 (hedonic pleasure orientation and hedonic relaxation orientation), 0.72 (hedonic pleasure orientation and eudaimonic orientation), and 0.42 (hedonic relaxation orientation and eudaimonic orientation). The mean scores were 4.78 (*SD* = 1.00) for hedonic pleasure orientation, 4.94 (*SD* = 1.01) for hedonic relaxation orientation, and 4.43 (*SD* = 1.06) for eudaimonic orientation.

### Measurement Invariance

As seen in Table 1, the gender invariance tests showed that the configural invariance (baseline) model was acceptable. Compared to the configural invariance model, ΔCFI and ΔRMSEA were below Chen’s (2007) cutoff for rejecting measurement invariance in the metric, scalar, and strict invariance models. Therefore, the three-factor structure of the Japanese HEMA scale showed configural, metric, scalar, and strict invariance across gender groups (see also Table 2 for factor loadings and inter-factor correlations).

**TABLE 1:** Fit indices for measurement invariance of the Japanese HEMA scale (11 items).

**Model**	**χ ^2^**	***df***	**CFI**	**RMSEA (90% CI)**	**SRMR**	****Δ** CFI**	****Δ** RMSEA**
**Gender invariance**							
Configural (structure)	527.64	82	0.936	0.076 (0.070, 0.082)	0.053	—	—
Metric (loadings)	546.47	90	0.935	0.073 (0.067, 0.079)	0.054	−0.001	−0.003
Scalar (intercepts)	577.12	98	0.931	0.072 (0.066, 0.078)	0.054	−0.005	−0.004
Strict (residuals)	564.75	109	0.935	0.066 (0.061, 0.072)	0.055	−0.001	−0.010
**Age invariance**							
Configural (structure)	624.90	123	0.931	0.080 (0.074, 0.087)	0.055	—	—
Metric (loadings)	655.82	139	0.929	0.077 (0.071, 0.083)	0.058	−0.002	−0.003
Scalar (intercepts)	707.55	155	0.924	0.075 (0.070, 0.081)	0.059	−0.007	−0.005
Strict (residuals)	730.78	177	0.924	0.070 (0.065, 0.076)	0.066	−0.007	−0.010

*CFI, comparative fit index; RMSEA, root mean square error of approximation; CI, confidence interval; and SRMR, standardized root mean square residual. Δ = difference. All χ^2^ values are significant at *p* < 0.001.*

**TABLE 2:** Strict invariant standardized factor loadings and factor correlations for the Japanese HEMA scale (11 items).

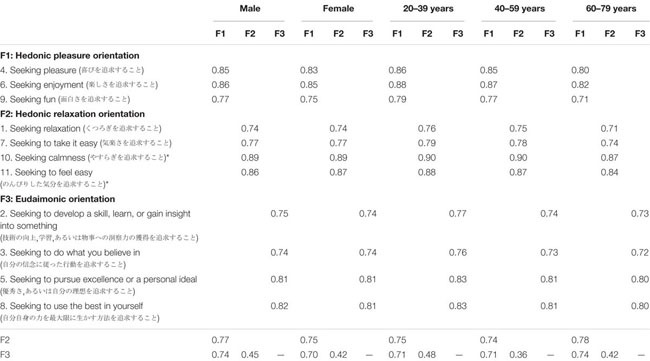

The Japanese translations are indicated in parentheses. *Added items for the Japanese version.

For the age invariance tests, the configural invariance (baseline) model was acceptable. Compared to the configural invariance model, ΔCFI and ΔRMSEA were below the cutoff for rejecting measurement invariance in the metric, scalar, and strict invariance models. Thus, the three-factor structure of the Japanese HEMA scale showed configural, metric, scalar, and strict invariance across age groups.

Note that the same patterns of results were obtained when we included Huta and Ryan’s (2010) original nine-item only. The three-factor structure of the original HEMA scale showed configural, metric, scalar, and strict invariance across gender and age groups (see Table 3 for invariance tests and Table 4 for factor loadings and inter-factor correlations).

**TABLE 3:** Fit indices for measurement invariance of the original HEMA scale (9 items).

**Model**	**χ ^2^**	***df***	**CFI**	**RMSEA (90% CI)**	**SRMR**	****Δ** CFI**	****Δ** RMSEA**
**Gender invariance**							
Configural (structure)	324.23	48	0.947	0.078 (0.070, 0.086)	0.049	—	—
Metric (loadings)	336.42	54	0.946	0.074 (0.067, 0.082)	0.050	−0.001	−0.004
Scalar (intercepts)	358.02	60	0.943	0.072 (0.065, 0.080)	0.050	−0.004	−0.006
Strict (residuals)	350.55	69	0.946	0.066 (0.059, 0.073)	0.052	−0.001	−0.012
**Age invariance**							
Configural (structure)	360.24	72	0.946	0.080 (0.072, 0.088)	0.050	—	—
Metric (loadings)	383.58	84	0.944	0.075 (0.068, 0.083)	0.054	−0.002	−0.005
Scalar (intercepts)	425.72	96	0.939	0.074 (0.067, 0.081)	0.057	−0.007	−0.006
Strict (residuals)	451.25	114	0.937	0.068 (0.062, 0.075)	0.064	−0.009	−0.012

*CFI, comparative fit index; RMSEA, root mean square error of approximation; CI, confidence interval; and SRMR, standardized root mean square residual. Δ = difference. All χ^2^ values are significant at *p* < 0.001.*

**TABLE 4:** Strict invariant standardized factor loadings and factor correlations for the original HEMA scale (9 items).

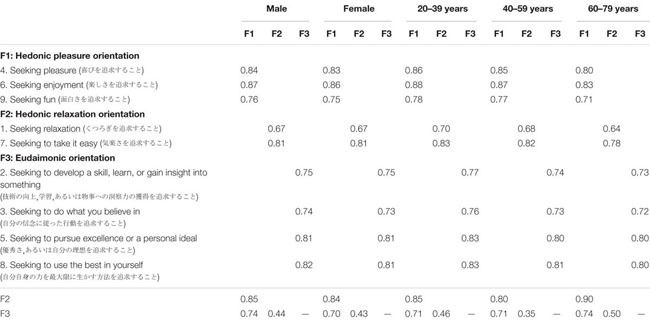

*The Japanese translations are indicated in parentheses.*

### Latent Mean Differences

Because strict invariance was established, we compared latent mean differences of the three Japanese HEMA subscales across gender and age groups (see also Supplementary Table S1 for descriptive statistics by gender and age groups). Gender differences showed less than small effect sizes (*d*s = 0.01–0.17). Age differences also showed less than small effect sizes (*d*s = 0.03–0.17).

The same patterns of results were found when we analyzed the original nine-item scale (Huta and Ryan, 2010). Gender and age differences for the three subscales showed less than small effect sizes (*d*s = 0.01–0.18 and 0.02–0.17, respectively).

### Internal Consistency, Temporal Stability, and Criterion Validity

Alpha and omega coefficients were 0.86 and 0.84, respectively, for hedonic pleasure orientation, 0.89 and 0.92, respectively, for hedonic relaxation orientation, and 0.86 and 0.88, respectively, for eudaimonic orientation. Test-retest ICCs were 0.55, 95% CI (0.51, 0.58) for hedonic pleasure orientation, 0.51, 95% CI (0.47, 0.54) for hedonic relaxation orientation, and 0.63, 95% CI (0.60, 0.66) for eudaimonic orientation (all *p*s < 0.001; see also Supplementary Table S2 for estimates by gender and age groups).

Correlations were observed between the three Japanese HEMA subscales and outcome variables (all *p*s < 0.001, unless otherwise noted; see also Supplementary Table S2 for estimates by gender and age groups). Hedonic pleasure orientation was strongly and positively correlated with positive affect [*r* = 0.34, 95% CI (0.30, 0.38)], and psychological well-being [*r* = 0.36, 95% CI (0.32, 0.40)], and was moderately and positively correlated with life satisfaction (*r* = 0.26, 95% CI [0.22, 0.30]) and interdependent happiness (*r* = 0.29, 95% CI [0.24, 0.33]). Hedonic relaxation orientation was weakly and positively correlated with life satisfaction [*r* = 0.14, 95% CI (0.10, 0.19)], positive affect [*r* = 0.18, 95% CI (0.14, 0.23)], psychological well-being [*r* = 0.12, 95% CI (0.08, 0.17)], and interdependent happiness [*r* = 0.15, 95% CI (0.10, 0.19)]. Eudaimonic orientation was strongly and positively correlated with psychological well-being [*r* = 0.50, 95% CI (0.46, 0.53)] and moderately and positively correlated with life satisfaction [*r* = 0.27, 95% CI (0.23, 0.31)], positive affect [*r* = 0.28, 95% CI (0.23, 0.32)], and interdependent happiness [*r* = 0.27, 95% CI (0.23, 0.31)]. Hedonic pleasure and eudaimonic orientations were weakly and negatively correlated with negative affect [*r*s = −0.14, 95% CIs (−0.18, −0.09)]; however, no association was found between hedonic relaxation orientation and negative affect [*r* = 0.01, 95% CI (−0.04, 0.05), *p* = 0.832].

## Discussion

Using an adult sample, this study investigated the measurement invariance and psychometric properties, particularly internal consistency, six-week temporal stability, and criterion validity, of the Japanese HEMA scale. We found evidence for configural, metric, scalar, and strict invariance for the three-factor structure of the scale across two gender groups as well as three age groups (20–39, 40–59, and 60–79 years). The effect sizes were less than small in latent mean differences of the scale across the demographic groups. In addition, these findings were consistent for the Japanese 11-item and the original 9-item scales. To our knowledge, this is the first report establishing the equivariance and showing latent factor means of the HEMA scale across gender and age.

The Japanese HEMA scale also indicated adequate internal consistency, temporal stability, and criterion validity in our sample. Aligning with previous research (Huta and Ryan, 2010; Asano et al., 2014, 2018; Braaten et al., 2019; see, for a review, Huta, 2016), we found high internal consistency and moderate temporal stability over a six-week period for all three subscales. These results suggest that the scale is a relatively reliable measure of the hedonic and eudaimonic conceptions of well-being as orientations. We also obtained results consistent with those of previous studies on criterion validity of the scale (Huta and Ryan, 2010; Asano et al., 2014, 2018; Braaten et al., 2019; see, for a review, Huta, 2016). Hedonic pleasure and eudaimonic orientations were associated with life satisfaction, positive affect, negative affect, and psychological well-being. Hedonic relaxation orientation showed associations with these outcome variables, except for negative affect. In addition, we initially demonstrated that all three subscales were associated with interdependent happiness. Our findings imply that the three orientations assessed by the Japanese HEMA scale relate to well-being as experiences and functioning derived from both Western and Eastern perspectives (Joshanloo, 2014; Uchida and Oishi, 2016).

Several limitations should be addressed. First, the current sample was recruited online and may not directly represent the Japanese adult population in terms of demographic and socioeconomic characteristics. Further research with nationally representative samples is necessary to test the generalizability of the findings on measurement invariance and latent means. Second, the participants were limited to those who lived in Japan. Future studies should examine measurement invariance of the original English and Japanese HEMA scale across nations. Third, although we assessed the Japanese HEMA scale at the trait-level representing a person’s typical or general orientations, the results were based on a short-term longitudinal study. More work needs to be done to examine the Japanese HEMA scale’s temporal stability over longer periods (years). Fourth, the study relied on cross-sectional self-report measures of outcome variables. It would be informative in future research to test the Japanese HEMA scale’s criterion validity with behavioral measures later in life, including academic achievement, job performance, and lifetime earnings.

Despite these shortcomings, we provided evidence regarding the factorial invariance, internal consistency, temporal stability, and criterion validity of the Japanese HEMA scale among adults. Our findings suggest that the Japanese HEMA scale is useful to capture the hedonic and eudaimonic conceptions of well-being as orientations. We hope that the present paper inspires further studies on well-being using the HEMA scale.

## Data Availability Statement

The datasets generated for this study are available on request to the corresponding author.

## Ethics Statement

The studies involving human participants were reviewed and approved by the Kurume University Institutional Review Board. The patients/participants provided their written informed consent to participate in this study.

## Author Contributions

RA developed conception and design of the study. RA collected the data and performed the statistical analysis. RA drafted the manuscript. TI and ST revised the manuscript.

## Conflict of Interest

The authors declare that the research was conducted in the absence of any commercial or financial relationships that could be construed as a potential conflict of interest.
