# Supplementary material for: The Hedonic and Eudaimonic Motives for Activities: Measurement Invariance and Psychometric Properties in an Adult Japanese Sample
Source: Front Psychol. 2020 Jun 10;11:1220. doi: 10.3389/fpsyg.2020.01220 (PMC7298781; doi:10.3389/fpsyg.2020.01220)
Supplement: Supplementary file 1 [file Data_Sheet_1.docx]

**Supplementary Material for**

**The Hedonic and Eudaimonic Motives for Activities:**

**Measurement Invariance and Psychometric Properties in an Adult Japanese Sample**

***Frontiers in Psychology***

**doi: 10.3389/fpsyg.2020.01220**

Table S1

*Means for the Japanese HEMA Scale by Gender and Age Groups*

|  | Male | Female | 20–39y | 40–59y | 60–79y |
| --- | --- | --- | --- | --- | --- |
| Hedonic pleasure orientation | 4.79 (1.03) | 4.78 (0.99) | 4.86 (1.08) | 4.78 (1.03) | 4.71 (0.89) |
| Hedonic relaxation orientation | 4.88 (1.00) | 4.99 (1.01) | 4.98 (1.05) | 4.99 (1.04) | 4.85 (0.93) |
| Eudaimonic orientation | 4.53 (1.03) | 4.35 (1.04) | 4.46 (1.11) | 4.44 (1.04) | 4.40 (1.02) |

*Note*. Values in parentheses indicate standard deviation.

Table S2

*Internal Consistency, Temporal Stability, and Criterion Validity for the Japanese HEMA Scale by Gender and Age Groups*

|  | Male | | |  | Female | | |  | 20–39y | | |  | 40–59y | | |  | 60–79y | | |
| --- | --- | --- | --- | --- | --- | --- | --- | --- | --- | --- | --- | --- | --- | --- | --- | --- | --- | --- | --- |
|  | HPO | HRO | EO |  | HPO | HRO | EO |  | HPO | HRO | EO |  | HPO | HRO | EO |  | HPO | HRO | EO |
| Cronbach’s alpha | 0.86 | 0.89 | 0.86 |  | 0.85 | 0.89 | 0.85 |  | 0.87 | 0.89 | 0.87 |  | 0.85 | 0.89 | 0.85 |  | 0.85 | 0.88 | 0.86 |
| McDonald’s omega | 0.85 | 0.90 | 0.89 |  | 0.83 | 0.93 | 0.88 |  | 0.85 | 0.93 | 0.89 |  | 0.84 | 0.91 | 0.87 |  | 0.83 | 0.92 | 0.89 |
| Test-retest ICC | 0.51 | 0.48 | 0.60 |  | 0.58 | 0.53 | 0.65 |  | 0.47 | 0.44 | 0.64 |  | 0.59 | 0.59 | 0.62 |  | 0.58 | 0.48 | 0.63 |
| **Outcome variables** |  |  |  |  |  |  |  |  |  |  |  |  |  |  |  |  |  |  |  |
| Life satisfaction | 0.24 | 0.15 | 0.26 |  | 0.28 | 0.14 | 0.28 |  | 0.17 | 0.06 | 0.22 |  | 0.34 | 0.21 | 0.29 |  | 0.33 | 0.20 | 0.33 |
| Positive affect | 0.36 | 0.22 | 0.31 |  | 0.34 | 0.14 | 0.26 |  | 0.32 | 0.14 | 0.27 |  | 0.40 | 0.22 | 0.28 |  | 0.37 | 0.24 | 0.30 |
| Negative affect | −0.18 | −0.06 | −0.17 |  | −0.09 | 0.05 | −0.09 |  | −0.11 | 0.05 | −0.15 |  | −0.21 | −0.02 | −0.16 |  | −0.16 | −0.09 | −0.14 |
| Psychological well-being | 0.37 | 0.15 | 0.51 |  | 0.35 | 0.11 | 0.48 |  | 0.33 | 0.06 | 0.52 |  | 0.38 | 0.09 | 0.49 |  | 0.44 | 0.29 | 0.51 |
| Interdependent happiness | 0.31 | 0.19 | 0.30 |  | 0.26 | 0.12 | 0.26 |  | 0.21 | 0.09 | 0.27 |  | 0.36 | 0.17 | 0.27 |  | 0.40 | 0.29 | 0.36 |

*Note*. ICC = intraclass correlation coefficient; HPO = hedonic pleasure orientation; HRO = hedonic relaxation orientation; EO = eudaimonic orientation.
